# Molecular Dynamics Simulation and Experimental Verification of the Interaction between Cyclin T1 and HIV-1 Tat Proteins

**DOI:** 10.1371/journal.pone.0119451

**Published:** 2015-03-17

**Authors:** Kaori Asamitsu, Takatsugu Hirokawa, Yurina Hibi, Takashi Okamoto

**Affiliations:** 1 Department of Molecular and Cellular Biology, Nagoya City University Graduate School of Medical Sciences, Nagoya, Aichi, Japan; 2 Molecular Profiling Research Center for Drug Discovery (molprof), National Institute of Advanced Industrial Science and Technology (AIST), Tokyo, Tokyo, Japan; Baylor College of Medicine, UNITED STATES

## Abstract

The viral encoded Tat protein is essential for the transcriptional activation of HIV proviral DNA. Interaction of Tat with a cellular transcription elongation factor P-TEFb containing CycT1 is critically required for its action. In this study, we performed MD simulation using the 3D data for wild-type and 4CycT1mutants3D data. We found that the dynamic structural change of CycT1 H2’ helix is indispensable for its activity for the Tat action. Moreover, we detected flexible structural changes of the Tat-recognition cavity in the WT CycT1 comprising of ten AAs that are in contact with Tat. These structural fluctuations in WT were lost in the CycT1 mutants. We also found the critical importance of the hydrogen bond network involving H1, H1’ and H2 helices of CycT1. Since similar AA substitutions of the Tat-CycT1 chimera retained the Tat-supporting activity, these interactions are considered primarily involved in interaction with Tat. These findings described in this paper should provide vital information for the development of effective anti-Tat compound.

## Introduction

Human immunodeficiency virus type 1 (HIV-1) currently infects an estimated 35.3 million people worldwide, and the numbers of infected people and death due to AIDS continue to rise despise the availability of antiviral drugs [1]. Since current anti-HIV-1 drugs mainly target viral protease and reverse transcriptase, selective drug pressure coupled with the high rate of HIV-1 infection and high mutation rate during each infection cycle quickly confer resistance to these drugs [2]. Thus, development of new anti-HIV-1 therapeutics targeting additional vial and cellular cofactors such as viral transcription that is essential for viral replication remains a pressing need.

Transcription from the integrated proviral DNA of HIV-1 is crucially regulated by a virus-encoded transcription factor Tat [3,4,5,6]. The Tat-mediated trans-activation of HIV-1 provirus requires an interaction among a cellular transcription factor, positive transcription elongation factor b (P-TEFb), Tat and TAR element, an RNA stem-loop structure specifically formed at the 5’-end of all HIV-1 mRNA transcripts [7,8,9]. P-TEFb contains a regulatory subunit cyclin T1 (CycT1) and a catalytic subunit cyclin-dependent kinase 9 (Cdk9) [9]. Tat recruits P-TEFb to the nascent viral transcripts, allowing Cdk9 to hyperphosphorylate the C-terminal domain (CTD) of RNA polymerase II (RNAPII), stimulates the transcriptional processivity of RNAPII and eventually activates viral transcription at the step of elongation [10,11,12,13].

Previous reports have revealed functional motifs of CycT1: within its polypeptide consisting of 726 amino acid (AA) residues that contains a cyclin box domain (AA positions 31 to 250), a coiled-coil sequence (from 379 to 530) and a PEST sequence (from 709 to 726) [13,14]. The first 272 amino acids of CycT1 were sufficient to bind Tat and TAR, and mediate Tat activation [7]. The central region of CycT1 250–272, termed the Tat-TAR recognition motif (TRM), is crucial for forming the Tat-CycT1-TAR ternary complex [8]. Within CycT1 TRM, Cys261 was considered essential because of its binding to Tat and TAR by forming a Zn^2+^-dependent interaction together with other Cys and His residues within Tat [8,10,11,15]. A number of studies using mutation analyses have revealed roles of various AA residues within CycT1, the functional integrity of TAR/Tat/P-TEFb complex and the molecular action of Tat. Besides TRM, there are other regions of CycT1 that are essential for the Tat-mediated transactivation: the N-terminal cyclin box involved in the Tat-mediated transcriptional activation by directly binding to Cdk9 [14,16,17,18].

Analysis of the crystal structure of the Tat/P-TEFb complex has revealed multiple hydrogen bonds in the interface between Tat and CycT1 N-terminus as well as within the CycT1 molecule [19]. In our previous report, we identified functionally crucial AA residues in the CycT1 N-terminal region [20]. We observed that Ala-substitution mutants derived from CycT1, namely Q46A, Q50A and F176A, abolished Tat activation. When such substitutions were introduced into the CycT1-Tat chimeric protein, the Q46A mutant among other mutants, behaved as a wild type, suggesting that Q46 might solely be involved in CycT1-Tat binding. These observations revealed a unique complex configuration among these AA residues in contact with Tat and facilitated us to further explore the functional integrity among these CycT1 AA residues in contact with Tat.

In recent years, molecular dynamics (MD) simulation of protein molecules have been adopted to further analyze the dynamic characteristics of proteins [21,22,23,24]. For example, spontaneous opening and reclosing of the HIV-1 protease flaps observed in NMR was reproduced by MD, and mutations of the critical AA residues abolished such dynamic fluctuation, which was correlated with the lack of catalytic action [22]. Furthermore, Miller et al. [23] deciphered the structural basis for the protein-protein interaction by MD simulation and confirmed by experimental approaches using AA substitution mutants.

In this study, we have evaluated the effects of these CycT1 AAs in the Tat-mediated transcriptional activation from HIV-1 long terminal repeat (LTR) by analyzing the structural flexibility and the functional activity of substitution mutants of these AAs. We found biological relevance of an intra- molecular hydrogen bond network within CycT1 as a crucial interface for the Tat-binding. Although various amino acid residues of CycT1 have been implicated in its specific binding to Tat including Q46, Q50, and F176, we have deciphered novel amino acid residues of CycT1, Q56, N60 and H183 that are involved in its Tat-binding through hydrogen bonding using MD simulation. In addition, MD simulation has revealed the dynamic changes of CycT1 local structure.

## Materials and Methods

### Molecular simulation

The 3D structure of wild type human CycT1 was extracted from the crystal structure of P-TEFb in complex with HIV-1 Tat (PDBID: 3MI9) [19] and MD simulations were carried out. Structural models for four CycT1 mutant proteins (Q46A, Q50A, Q46AQ50A and F176A) were constructed based on the structural coordinate of wild type human CycT1 by homology modeling using Molecular Operating Environment (MOE) (Chemical Computing Group Inc.).

MD simulations were carried out using the AMBER ver.11 package with the ff99SB force field [25]. SGI Rackalbe RP2 Standard-Depth Servers C2108-RP2 (Intel Xeon Processor E5–2670, 16CPU/node) at AIST/molprof was used as the computational hardware in this simulation. The protein structure was surrounded with a 15 Å layer of TIP3PBOX water molecules. The electrostatic charge was neutralized by adding counter ions using the LeaP program of AMBER ver.11. After minimization, heating and equilibration, the production MD phase was carried out at 300K for 100 ns with a time step of 1 fs using the constant volume and temperature (NVT) ensemble and the Particle Mesh Ewald algorithm for the calculation of electrostatic interactions [26]. The initial velocity of atoms was generated at 100K on heating phase with a Maxwellian distribution and maintained. The pressure was kept at 1 bar by Berendsen weak-coupling approach during equilibration [27]. Coordinates are recorded every 10 ps. The simulation trajectories were then analyzed for the sampled conformations in all structural models. The obtained trajectory was processed utilizing the AMBER11 tool ptraj for the calculations of RMSD and PMSF of proteins.

### Principal Component Analysis (PCA)

Principal Component Analysis (PCA) was performed on the 3D coordinates Cα atoms of the Tat Recognition Residues (TRR) of CycT1 for both native and mutant MD trajectories. The PCA results of native and mutant structures were analyzed and visualized the conformational differences by plotting to PC1 (primary component 1) and PC2 axes. PCA is a standard statistical method routinely used to identify variable correlations in a system from atomic fluctuations in a MD trajectory [28,29,30]. The distance matrix of root mean square deviation (RMSD) of TRR Cα atoms for the combined trajectories of native and mutant structures was calculated after the structure alignment of full Cα atoms for preparation of input matrix to PCA. PCA was performed using R (version 3.1.1.) which is a software language and environment for statistical computing and graphics.

### Other software for the analysis of protein structure

All figures displaying the protein structure were prepared with PyMol software (http://pymol.soucefoge.net). Protein contact analysis was performed by using MOE-ContactAnalyzer subroutine equipped in MOE which implements the hydrogen bond test developed by Stickle and Rose [31].

### Plasmids

The luciferase expression plasmid under the transcriptional control of HIV-1 LTR, CD12-luc (containing HIV-1 LTR U3 and R) [32] and the expression plasmid for the FLAG-tagged CycT1 protein (pcDNA-CycT1 (1–278) adding the FLAG-tag to the N-terminus of CycT1 (1–278) AA as described [20]) have been described previously. For expression of the Myc-tagged CDK9 protein, the Myc-tag was first introduced into pcDNA3.1 by synthesized oligonucleotides (sense:5’-agctagcaccatggaacaaaaactcatctcagaagaggatctgaagcttat-3’; anti-sense: 5’-ataagcttcagatcctcttctgagatgagtttttgttccatggtgctagct-3’) by using the NheI-HindIII sites of this plasmid and full-length CDK9 cDNA was then inserted in-frame to the downstream of this plasmid. The CDK9 cDNA fragment was amplified by PCR using pSVRevCDK9 [33] as a template with the CDK9-specific primers (sense primer: 5’-ggggatccatggcaaagcagtacgactcggtggag-3’; anti-sense primer: 5’-gcggatcctcagaagacgcgctcaaactccgtc-3’). The expression plasmids for HA-Tat (pcDNA-Tat) [34, 35] and CycT1-Tat fusion protein (pLINK-CycT1-Tat) [36] were kind gifts from Drs. B. M. Peterlin and K. Fujinaga. Various Cyclin T1 mutants were then constructed based on pcDNA-CycT1(1–278) or pLINK-CycT1-Tat by using oligonucleotides that contain degenerated nucleotides at positions for Ala using Quick- change mutagenesis kit (Stratagene, La Jolla, CA). All mutant constructs were analyzed by dideoxynucleotide sequencing to ensure that the proper mutations were present before further experiments.

### Luciferase Reporter Assay and determination of protein expression level

A murine NIH3T3 cell line, where Cys261 is substituted to Tyr261 and not susceptible for the Tat-mediated trans- activation from HIV-1 LTR, were transfected with CD12-luc (80ng) and CycT1 plasmids (20ng) in the presence of pcDNA-Tat (40ng) or plasmids for CycT1-Tat chimeras (40ng) with Fugene6 (Roche) [20]. Twenty-four h after transfection, cells were lysed and subjected to determination of luciferase activity (Promega). To assay the expression of these Cyclin T1 constructs, HEK293 cells were transfected with 1.0 μg of each of these Flag-tagged Cyc T1 constructs with or without 1.0 μg of HA-tagged Tat or Myc-tagged CycT1-Tat chimera constructs by using calcium-phosphate-mediated transfection. Forth-eight hours after transfection, cells were harvested and equal amounts of protein from cell lysates were separated by SDS-PAGE. Protein expression level was determined by Western blotting using specific antibodies.

### Co-immunoprecipitation

HEK293 cells were transfected with plasmid DNA constructs using polyethylenimine MW 25000 (Polysciences). Twenty-four hours after transfection, cells were lysed in high salt lysis buffer (50mM Tris-HCl pH 8.0, 300mM NaCl, 1mM EDTA, 0.5% NP-40, 10% glycerol) supplemented with protease inhibitor cocktail (Roche). Each total cell lysate was divided into two samples. One sample was immunoprecipitated with anti-M2 beads (Sigma) for 4hr at 4°C. The other was incubated with anti-HA antibody for 4hr and then added protein G-agarose (GE Healthcare) for an additional 2 hr at 4°C. The beads were washed three times with cell lysis buffer, and immunoprecipitates were eluted by boiling the beads for 3 min in 2x sodium dodecyl sulfate (SDS) sample buffer.

### Immunoblotting

Proteins were resolved by SDS-PAGE and transferred to Immobilone-P (Millipore). Membranes were immunoblotted with the indicated antibodies and the bound antibodies were visualized with horseradish-peroxidase-conjugated antibodies against rabbit or mouse IgG (GE Healthcare) using SuperSignal West Pico Chemiluminescent substrate (Piace).

### Antibodies

The following commercial antibodies were obtained from the indicated suppliers: anti-FLAG antibody (SIGMA), antibody to human α-tubulin (Santa Cruz Biotechnology), anti-Myc antibody (MBL, Nagoya, Japan) and anti-HA antibody (Santa Cruz Biotechnology).

## Results

### CycT1 AA residues involved in Tat binding

**Fig 1:**
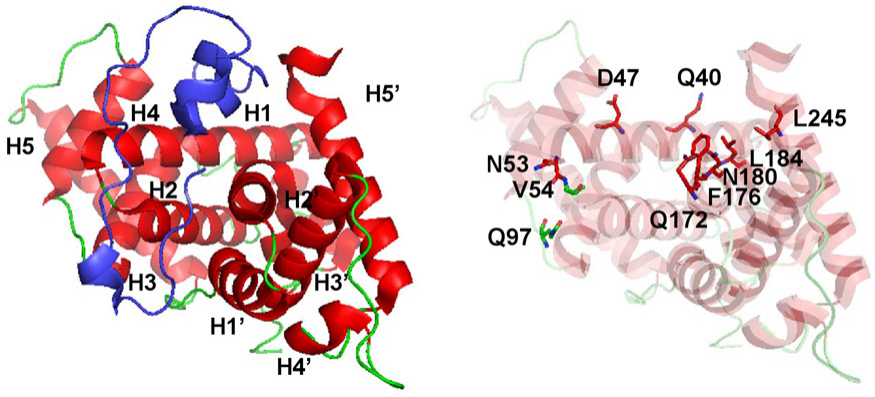
Characteristic α-helices of CycT1 (3MI9) and Tat recognition residues (TRR). (a) (Left panel) Crystal structure of CycT1-Tat complex obtained in the Tat/P-TEFb complex (PDBID:3MI9 [19]). Tat (blue) and Cyc T1 (red and green) are demonstrated in different colors. The locations of each α-helices are indicated: H1, 31–52aa; H2, 54–69aa; H3, 80–64aa; H4, 101–102aa; H5, 125–143aa; H1’, 153–163aa; H2’, 168–184aa; H3’, 193–207aa; H4’, 221–224aa; H5’, 231–245. (Right panel) Locations of Tat recognition residues in CycT1: Q40, D47, N53, V54, Q97, Q172, F176, N180, L184 and L245 (which side chains are shown in stick).

The crystal structure of trimolecular complex consisting of CycT1, Cdk9 and Tat has recently been resolved by Tahirov et al [19]. They revealed that Tat forms partial α-helical structures upon binding to CycT1 and inserts itself into a cleft formed by H1, H1’ and H2’ of CycT1 [19]. To identify amino acid residues involved in the interaction between Tat and CycT1, we performed the protein contact analysis using ContactAnalyzer option in MOE that can search inter-residue hydrogen bonds, ionic contacts, disulfide bonds and hydrophobic contacts. ContactAnalyzer implements the hydrogen bond test developed by Stickle and Rose [31], which is sufficiently strict in isolating genuine hydrogen bonds. Thus, we detected ten amino acid residues of CycT1, Q40, D47, N53, V54, Q97, Q172, F176, N180, L184 and L245 in contact with Tat (Fig. 1) and named them as Tat recognition residues (TRR). In the 3D structural model of the CycT1-CDK9-Tat complex (3MI9) [19], the TRR is uniquely involved in the CycT1-Tat but not CycT1-CDK9 interaction. The details of TRR and their interaction modalities are described in Table 1. Then we found CycT1 mutants CycT1Q46A, CycT1Q50A and CycT1Q46AQ50A losing the Tat-supporting activity as well as the Tat-binding activity [20]. However, these AA residues were not included in TRR. In addition, although Q40, D47 and N53 residues belonged to the TRR, their Ala-substitution mutants did not lose the Tat-supporting activity. Thus, the direct physical contact of each TRR is not necessarily the prerequisite for its involvement in the functional association with Tat. In order to analyze the mechanism by which CycT1 recognizes Tat and support its action as a trans-activator of transcription from HIV-1, we performed MD simulation of CycT1 and its mutants. In order to predict 3D structures of the above CycT1 mutants, we adopted homology modeling using MOE (data not shown).

**Table 1:** Various chemical bonds involved in the Tat-CycT1 binding detected by ContactAnalyzer (MOE).

	type	chain	Pos Residue	chain	Pos Residue
1	HB	CycT1	Q40, NE2	Tat	S46, O
2	HB	CycT1	D47, OD1	Tat	Y47, OH
3	HB	CycT1	N53, OD1	Tat	Q17, N
4	HB	CycT1	V54, O	Tat	S16, OG
5	HB	CycT1	Q97, NE2	Tat	H13, O
6	HB	CycT1	Q172, NE2	Tat	M1, O
7	HB	CycT1	N180, OD1	Tat	Q35, NE2
8	HYD	CycT1	F176, CE1	Tat	M1, CE
9	HYD	CycT1	F176, CZ	Tat	F38, CD2
10	HYD	CycT1	L184, CD2	Tat	I39, CD1
11	HYD	CycT1	L184, CD1	Tat	L43, CD1
12	HYD	CycT1	L184, CD2	Tat	I45, CD1
13	HYD	CycT1	L245, CD2	Tat	L43, CD1

The chemical bonds between these two protein molecules within the distance of 4.5Å for hydrophobic cutoff as well as hydrogen bonds identified by MOE ContactAnalyzer are described here. There is no ionic and disulfide interaction within the distance of 4.5 Å and 2.5 Å, respectively. HB, hydrogen bond; HYD, hydrophobic interaction.

### MD simulations of various CycT1 proteins

**Fig 2:**
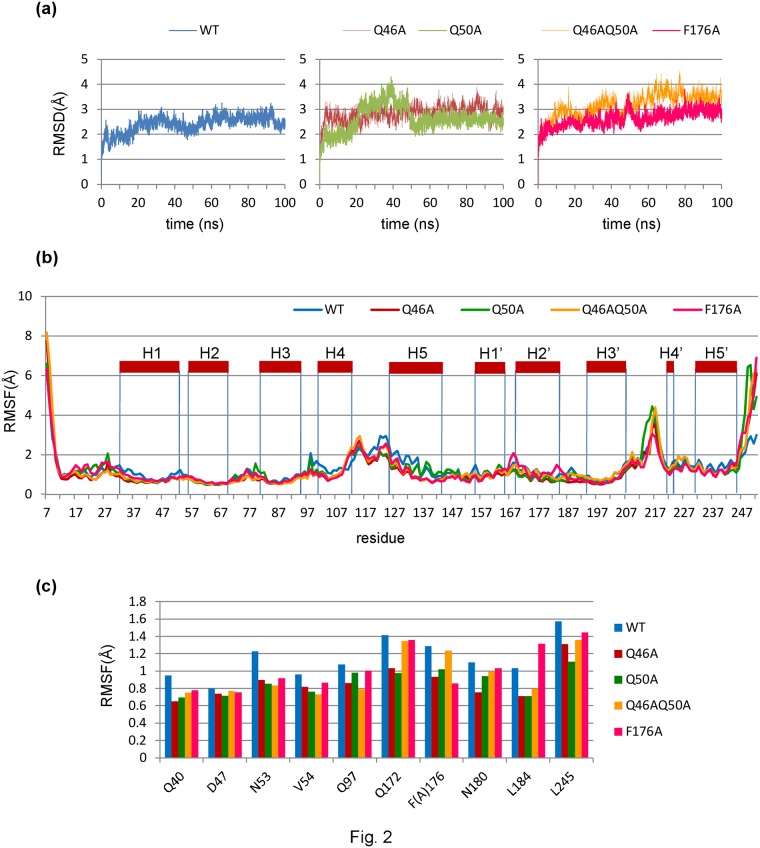
Molecular dynamics (MD) simulation. (a) Time course of root mean square deviations (RMSDs) of wild-type CycT1 (left) and CycT1 mutants (Q46A and Q50A (middle) and Q46AQ50A and F176A (right)). (b) Root mean square fluctuations (RMSFs) of CycT1 molecules. (c) RMSF of TRR residues.

We then performed MD simulation using AMBER ver. 11 as described in Experimental Procedures. We have carried out computation of MD simulation for 100 ns and reached the plateau of dynamic structural changes as shown in Fig. 2a (left panel). It was clearly demonstrated that the CycT1 structure could be readily stabilized alone without associating molecules such as Cdk9 and Tat. Similar MD computation of the predicted structural models of each CycT1 protein revealed structural stabilization within 100 ns. These findings suggest that each CycT1 protein is structurally stable by itself.

Atomic fluctuations (root mean square of fluctuation; RMSF) for each CycT1 model were analyzed. As shown in Fig. 2b, relative spatial fluctuation of H4 and H5 helices was detected for the wild type (WT) CycT1 over its mutants although the H4-H5 junction was similarly flexible among these CycT1 models. Interestingly, as these helices are located in the opposite surface of CycT1 in binding to Tat (Fig. 1) and not directly involved in Tat recognition, Q46A, Q50A and F176A amino acid substitutions might cause the structural stabilization of CycT1 protein and the relatively instable structure of WT CycT1 might be crucial for the recognition of Tat as previously reported with other proteins [37,38,39].

When the RMSF values of each TRR were compared (Fig. 2c), only a few AAs in WT CycT1, such as N53, exhibited remarkable fluctuation, suggesting that the loss of activity of CycT1 in supporting the Tat-mediated HIV transcription cannot be ascribed to a sole amino acid residue but rather to structural integrity of WT CycT1 protein. Thus, we performed principal component analysis (PCA), one of the multivariate analyses for analyzing the net effect of phenomena consisting of numerous factors, of the structural coordinate of TRR in each CycT1 model.

### PCA analyses of the MD simulation data for each CycT1 model

**Fig 3:**
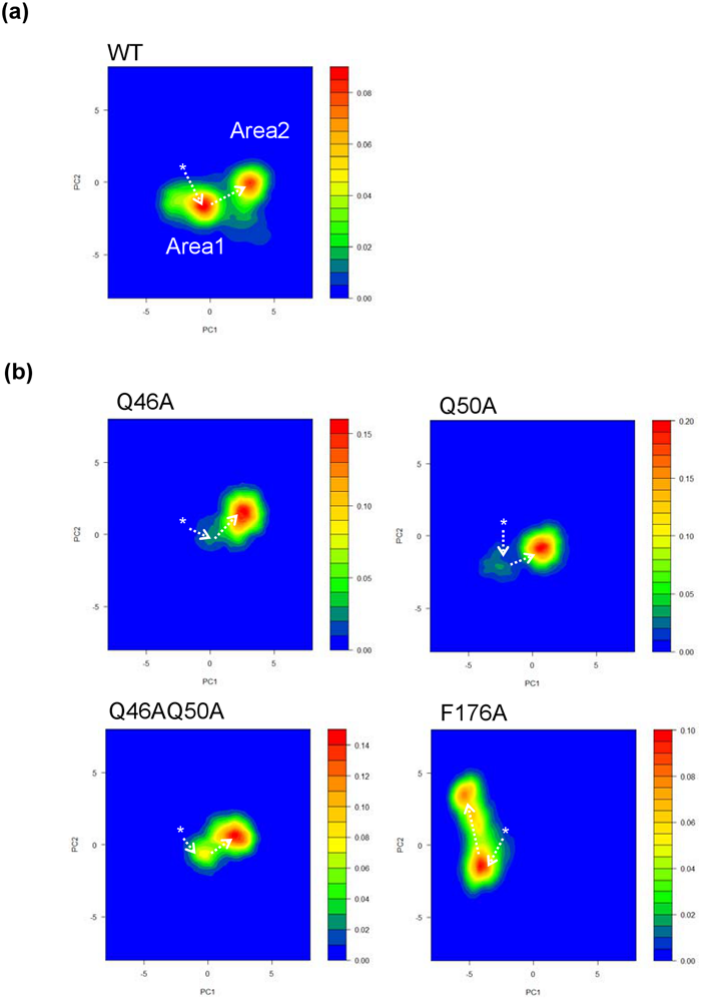
Two-dimensional PCA projections of trajectories obtained from MD simulations for various CycT1 proteins. Initially, PCA calculations were carried out with all the CycT1 models including WT, and CycT1 mutants, Q46A, Q50A, Q46AQ50A and F176A, in order to obtain the coordinate for each component. Then, using the rmsd distance data for Cα atoms of TRR obtained from this coordinate, each trajectory data set was plotted in accordance with principal components 1 and 2 for abscissa and ordinate, respectively. 2-D (primary component 1 (PC1)-PC2) plots of trajectories obtained with WT (a) and various CycT1 molecules (b). The position of 3MI9 CycT1 is indicated as “*”. Probability distribution (right) of MD trajectories based on PCA was estimated using kernel density estimation method in R version 3.1.1.

Initially, PCA calculations were carried out with all the CycT1 models including WT, and CycT1 mutants, Q46A, Q50A, Q46AQ50A and F176A. Then, using the RMSD distance data

for Cα atoms of TRR obtained from this computation, each trajectory data set was plotted in accordance with principal components 1 and 2 for abscissa and ordinate, respectively. Fig. 3a demonstrates the 2-dimensional scatterplot of PCA data obtained for the Cα atoms of TRR in WT CycT1. Similar plots were obtained for those in CycT1 mutants, Q46A, Q50A, Q46AQ50A and F176A (Fig. 3b). As demonstrated here, the PCA scatterplot of WT CycT1 revealed two distinctive structural clusters, namely “Area 1” and “Area 2” (Fig. 3a). There appears to be step-wise temporal transition from the crystal structure (3MI9; demoted as “*”) to Area 1 and finally to Area 2. However, when critical AA residues were substituted, only one major cluster remained and the other clusters were not clearly present (Q46A, Q50A and Q46AQ50A mutants) (Fig. 3b), suggesting the loss of flexibility by AA substitution. The PCA plot of F176A mutant exhibited aberrant appearance of Area 1and additional clusters, suggesting introduction of stable protein configuration by F176A substitution as well. Together with the data in Fig. 2c, fluctuations of TRRs seem to appear in somewhat coordinated fashion.

### Comparison of representative structures of various CycT1 models obtained by MD simulation

**Fig 4:**
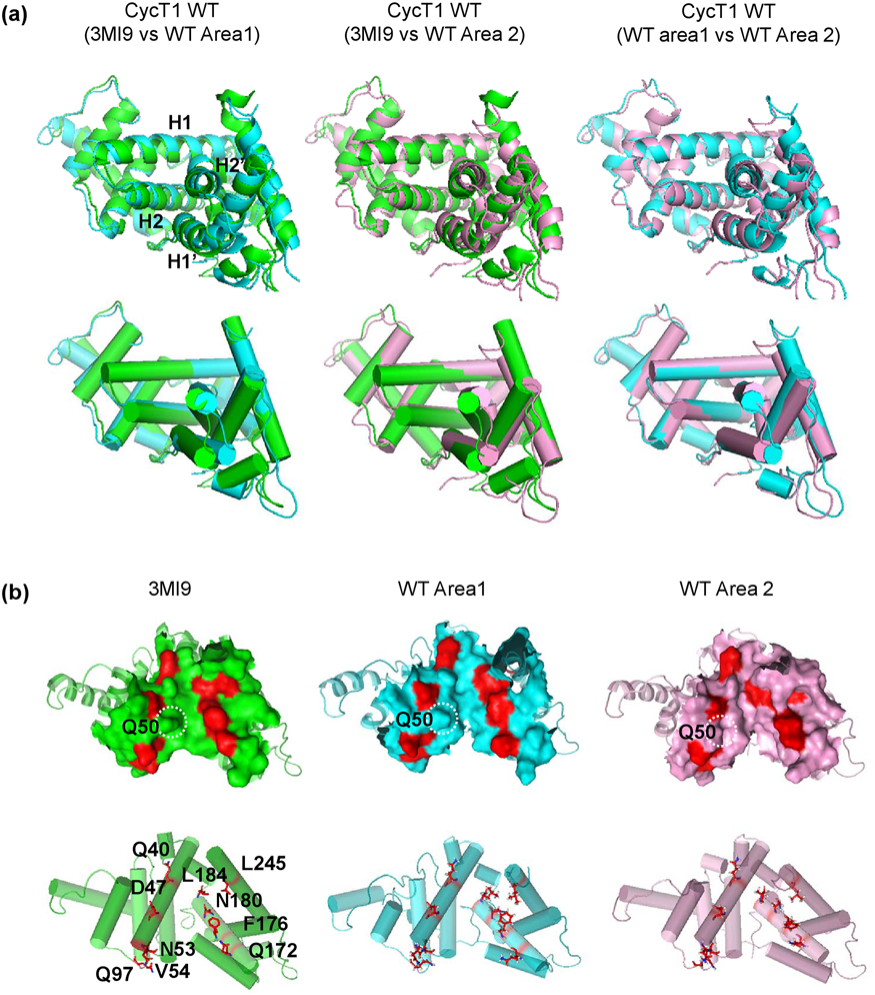
Comparison of representative structures of various CycT1 models obtained by MD simulation. (a) Comparison of CycT1 WT structures obtained by crystallographic analysis ((3MI9); green) and the representative structures predicted by MD simulation (Area1; cyan, Area 2; pink). Note the structural shifts of H1, H2, H1’ and H2’ helices upon superimposition. (b) The structural changes of Tat binding cavity circumscribed by TRR (red). Surface of TRR are shown in red and surrounded amino acids are shown in green (3MI9), cyan (Area 1) and pink (Area 2) in accordance with the color used in Fig. 4a. The position of Q50 is indicated by dotted circle. Lower panels indicate the positions of TRR with cylindrical helices for the backbone.

In order to compare representative 3D structures of CycT1 WT Areas 1 and 2 with crystallographic data (3MI9), 3D structures of these structural models were superimposed (Fig. 4a). Fig. 4a (left and center) shows that the H1 and H2 helices were longitudinally shifted and the H1’ and H2’ helices appeared to open outwardly in Area 1 and 2. When structures of Area 1 and 2 were compared (Fig. 4a, right), H1’ helix of Area 2 was shifted slightly inward although H2’ structure appeared stable, thus holding a cavity for Tat binding even in the absence of Tat.

We have further investigated the structural changes of Tat binding cavity circumscribed by TRR (Fig. 4b; red). It appears that the Tat binding cavity in CycT1 WT Area 1 (Fig. 4b, compare left and center) is expanded and then contracted as in Area 2 (Fig. 4b, right), which might be induced by the interaction between F176 and N180 followed the inward shift of the side chains of N180 and L184 that are within H2’ helix. In addition, the side chain of Q50, though not in a member of TRR, extruded upon Tat binding for further stabilization of the CycT1-Tat complex. Since AA substitutions at Q46, Q50 and both Q46 and Q50 did not appear to exhibit such structural changes upon MD simulation (Fig. 3), loss of such structural fluctuations might be necessary for Tat binding, thus the quasi-stable structure in transition might be a prerequisite for such action. In F176A CycT1 mutant (Fig. 3), where a central AA within the hydrophobic patch that is involved in the direct interaction with Tat was substituted, the dynamic structural changes was not directed to the Tat binding.

### Hydrogen bonds involved in the CycT1 intra- and inter-molecular helical interactions

We have previously reported the indispensability of CycT1 Q46 in Tat-binding [20]. Since Q46 appears to play a crucial role in inter-helical hydrogen bonding within CycT1, we have investigated hydrogen bond formation within CycT1 upon MD simulation. Table 2 shows the AA residues involved in such hydrogen bonding during MD simulation of WT and mutant CycT1 proteins. Three inter-helical interactions and two intra-helical interactions were identified in CycT1 WT (greater than 50% probability upon MD simulation). No aberrant hydrogen bonding was detected with CycT1 mutants (data not shown). However, minor inter-helical hydrogen bonding within WT CycT1 such as H1’-H2, H1-H2’ and H4-H5 became dominant (greater than 50% probability) upon AA substitutions (see Table 2 for the detail). It is noted that the bidirectional inter-helical interactions of H1: H2 involving Q46 and Q56 were observed in WT CycT1. We also detected a hydrogen bonding between H2 (N60) and H2’ (H183) in WT CycT1. When either Q46 or Q50 was substituted by Ala, hydrogen bonding involving the AA residues left unchanged was observed although to a lesser extent. These finding suggest the robustness of such hydrogen bonding in holding CycT1 structure. However, when both Q46 and Q50 were mutated, there was no longer any interhelical hydrogen bonding. In addition, when F176 was substituted by Ala, the hydrogen bonding between H2 and H2’ was abolished and that of H1 and H2 was significantly affected. The ability to form these hydrogen bondings appear to correlate with the CycT1’s ability to support the Tat-mediated transactivation of HIV transcription. These findings suggest that MD simulation could uncover the effect of subtle AA substitution on the structural integrity of CycT1 protein that is crucial for its biochemical action. These findings prompted us to examine the significance of these AA residues on the biological and biochemical effects of AA substitution of such residues.

**Table 2:** Inter and intra-helical hydrogen bonds within each CycT1 model.

			% appearance rate in each CycT1 model				
	donor	acceptor	WT	Q46A	Q50A	Q46AQ50A	F176A
inter-helical							
H2-H2'	N60, OD1	H183, NE2	**75.77**	44.56	0	0	0
H1-H2	Q46, OE1	Q56, NE2	**71.28**	-	44.5	-	**65.31**
H1-H2	Q56, OE1	Q46, NE2	**59.52**	-	36.64	-	**52.72**
H1-H2	H67, ND1	R38, NH1	37.29	35.18	41.56	**55.49**	11.68
H1'-H2	H154, NE2	N60,ND2	27.14	0	**79.91**	20.57	35.62
H1-H2'	Q46, OE1	H183, NE2	9.3	0	0	0	**50.07**
H4-H5	H109, ND1	Q129, HE21	12.57	21.13	44.35	**52.68**	21.58
inter-helical							
H2-H2	S55, OG	T58, OG1	**73.45**	**94.14**	**87.62**	**95.47**	**90.48**
H2-H2	N60, OD1	Q56, NE2	**61.83**	21.98	**53.88**	37.59	**65.89**
H1-H1	E34, OE1	R38, NH2	31.68	**56.99**	**66.93**	**84.57**	34.7
H1-H1	E34, OE1	R38, NE	10.51	**57.1**	48.24	40.65	0
H1-H1	E34, OE2	R38, NE	9.76	**68.38**	**81.53**	5.95	10.27

Appearance rate with the probability of 50% or greater during MD simulation is shown in bold.

### Functional significance of hydrogen bond network of CycT1 involving Q46 and Q56, N60 and H183

**Fig 5:**
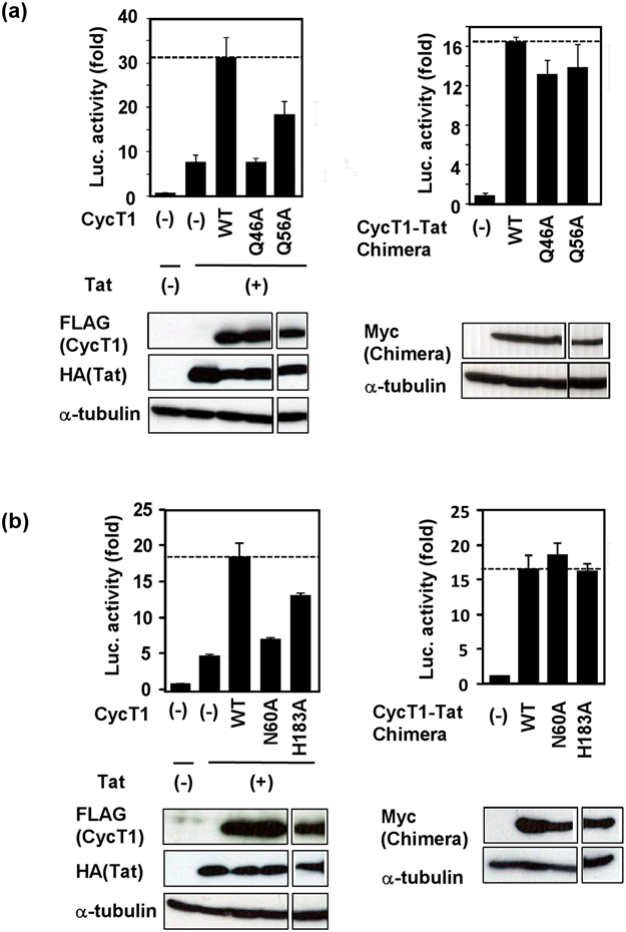
The transcriptional activity of CycT1 molecules in supporting the Tat-mediated HIV trans-activation: involvement of intra-molecular hydrogen bonds within CycT1, Q46:56 and N60:H183, forming the intra-molecular hydrogen bond network. (a) Effects of Ala-substitution in CycT1 on the Tat-transactivation (CycT1 Q46A and Q56A mutants). (b) Effects of Ala-substitution in CycT1 on the Tat-transactivation (CycT1 N60A and H183A mutants). Transcriptional activities of Tat co-transfected with CycT1 (left panel) or those of CycT1-Tat chimera (right panel) are shown. Western blots shown below indicate that the equivalent amount of each protein was expressed in the transfected cells. These protein bands were originated from the same blot for each panel.

It appears that intra-molecular hydrogen bond network among Q46, Q56, N60 and H183 determines the transcriptional activity of CycT1 molecule for the Tat action. In order to further examine the role of this hydrogen bond network, we created substitution mutants of these AAs and examined their effects on the Tat-mediated trans-activation of HIV-1 transcription. As shown in Fig. 5a (left panel), CycT1 Q56A substitution significantly lost its action on Tat activity. In addition, CycT1 Q46A mutant completely abolished the Tat-supporting activity, thus confirming our previous report [20]. These findings indicate the importance of double side-chain interactions of Q46 (Table 1). Interestingly, however, when these AA substitutions were introduced within the CycT1-Tat chimeric protein, only marginal effects were observed with Q46A and Q56A (Fig. 5a, right panel). These findings suggest that the dual hydrogen bonding between Q46 and Q56 is crucial for the protein-protein binding between CycT1 and Tat.

Similarly, Fig. 5b indicates that N60 and H183 AA residues are crucial for the Tat-supporting activity of CycT1. As shown in Fig. 5b (left), whereas CycT1N60A greatly lost the Tat-transactivation, CycT1H183A substitution did not remarkably exhibit deleterious effect when CycT1 and Tat were separately transduced. However, the effect of N60A and H183A substitutions, introduced in the CycT1-Tat fusion protein, did not affect the transcriptional activity (Fig. 5b, right).

**Fig 6:**
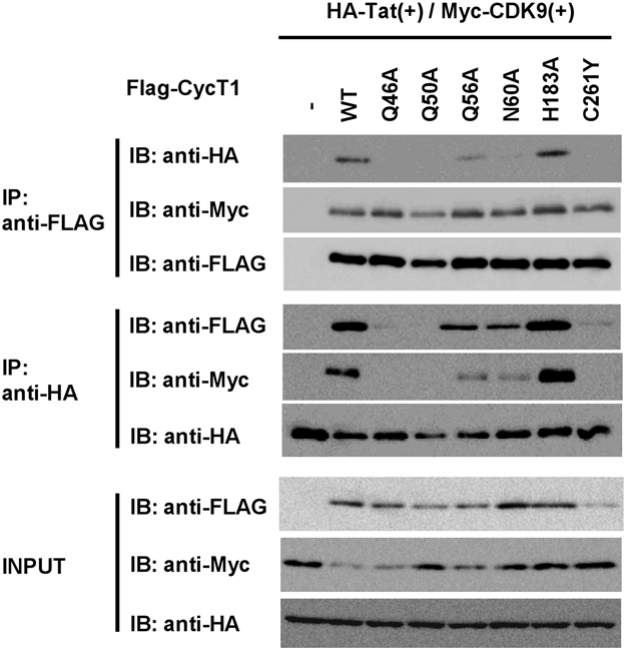
Effects of Ala-substitutions within Cyc T1 on the binding to Tat and CDK9 by immunoprecipitation. HEK293 cells were transfected with various CycT1 mutants (FLAG-tagged), HA-tagged Tat and Myc-tagged CDK9. The cell extracts were immunoprecipitated with anti-FLAG(M2) beads or anti-HA antibody. The immunoprecipitated complex was then immunoblotted with indicated antibodies. Expression of FLAG-CycT1, HA-Tat and Myc-CDK9 proteins in the transfected cells was confirmed by immunoblotting with indicated antibodies (“Input” panels).

To confirm that these mutants indeed lose the association with Tat without affecting its binding to Cdk9, we examined whether Cyc T1 mutants bind to Tat and CDK9 by immunoprecipitation. Those CycT1 mutants (FLAG-tagged) with Ala-substitution at Q46, Q56, N60 and H183 were transfected with Tat (HA-tagged) and CDK9 (Myc-tagged) expressing plasmids. When CycT1 proteins were immunoprecipitated, co-immunoprecipitated Tat and CDK9 were detected by Western blotting with anti-HA and anti-Myc antibodies, respectively (Fig. 6, upper two panels). We have previously reported that Q50A and C261Y CycT1 substitution mutants lost the Tat-binding [8,20]. In this study, we found that Q46A, Q56A and N60A CycT1 mutants similarly lost the Tat-binding. However, H183A mutant still exhibited Tat-binding activity, which was consistent with the fact that this mutant partially lost the effect on Tat-mediated transactivation (Fig. 5B). This is presumably due to the formation of intramolecular hydrogen bond of CycT1 involving 3 AAs: N60 and H154 as well as H183 (Table 2). In contrast, CDK9 was co-immunoprecipitated with these CycT1 mutants. WT CycT1 and its mutants showed similar affinity to FLAG-affinity beads (3^rd^ panel). When Tat was immunoprecipitated, on the other hand, co-immunoprecipitation of these CycT1 mutants were similarly observed (compare 1^st^ and 4^thrd^ panels). The amount of CDK9 co-immunoprecipitated with Tat was similar to the amount of various CycT1 mutants, presumably indicating that co-immunoprecipitation of CDK9 was mediated by CycT1 (5^th^ panel). Similar amounts of Tat were immunoprecipitaed by anti-HA antibody in cells transfected with these CycT1 proteins (6^th^ panel). These observations are consistent with the effects of various CycT1 mutations on the Tat-mediated transactivation: when CycT1-Tat chimeric proteins, wild-type and mutants, were used, Tat-mediated HIV-1 transactivation was restored. Interestingly, however, although CycT1 H183A mutant restored Tat-binding activity, it significantly, but not completely, impaired Tat-mediated trans-activation.

## Discussion

Tat is specifically and essentially required for the explosive replication of HIV-1, playing a critical role in aggressive viral transmission and emergence of drug-resistant viral isolates that are resistant to anti-HIV drugs as well as host immune responses. Thus, elucidation of the molecular mechanism by which Tat activates HIV transcription in the infected cells is crucial for developing a novel anti-HIV therapy against the Tat-CycT1 axis as a molecular target. In this regard, the structural analysis of dynamic Tat-CycT1 interaction and the detailed mapping are crucial for understanding the Tat-mediated transactivation.

This is the first report of MD simulation for CycT1 in binding to Tat, where we have analyzed the protein-protein interaction between CycT1 and Tat in order to further elucidate the molecular recognition mechanism. MD simulation has been used to depict a molecular mechanism by which bio-molecules exhibit their biological functions such as catalytic actions of HIV-1 protease [21,22], where MD simulation of HIV-1 protease the dynamic closing of the flap structure laced in the active site of viral protease was shown to be induced by the substrate peptide using MD simulation. In another example, MD simulation has deciphered the dynamic interaction between two tumor-related molecules, VHL and hypoxia-inducible transcription factor 1α (HIF1α) where distant two VHL regions are involved in its binding to HIF1α [23]. As in our study, Miteva et al [24] confirmed the findings obtained with site-directed mutagenesis by MD simulation.

Our findings with MD simulation of WT and mutant CycT1 proteins have been successful in elucidation of the structural fluctuation of the contact surface of each CycT1 protein upon Tat binding. We found the following: (i) the H1’ and H2’ helices in WT CycT1 were shifted outward upon MD simulation. However, defective CycT1 mutants containing AA substitution of either Q46A, Q50A, Q46AQ50A or F176A exhibited significantly reduced protein flexibility; (ii) a successive structural transition from the crystal structure (3MI9) to two distinct structures (“Area 1” and “Area 2”; Fig. 3) appears to be required for its stable interaction with Tat; (iii) the H1, H2 and H2’ helices of CycT1 are involved in the structural integration of CycT1 protein and are essential for its Tat-binding activity, which was confirmed by wet experiments; and (iv) abrogation of these intra-molecular hydrogen bonding could abolish the Tat-mediated HIV-1 gene expression. In fact, IP-WB experiments in this study (Fig. 6) have revealed diminished binding affinity between Tat and CycT1. It appears that the presence of localized cavity formed by these interacting helices should be crucial for the interaction with Tat and the Tat-mediated transactivation. Therefore, it is postulated that small molecular compounds that can preoccupy this Tat-binding cavity or allo-sterically alter the protein flexibility preferring the significant structural change in the Tat-binding cavity should conceptually inhibit its binding to Tat, thus robustly inhibiting HIV replication by blocking the Tat-mediated transcriptional activation.

The appearance of TRR fluctuation in relatively coordinated fashion for WT CycT1 (Figs. 2 and 3) has supported a possibility that the Tat-binding structure might have preexisted before the participation of Tat. In this context, our present findings appear to prefer the preexisting equilibrium model proposed by Goh et al [40] for the interaction between CycT1 and Tat since MD simulation (Fig. 3) has revealed the presence of intermediate state for its binding to Tat: upon MD simulation immediately after Tat removal, there were two distinct clusters of transient intermediate structures, Area 1 and Area 2, where the structure of Tat-binding cavity appeared to be expanded followed by contraction. Since in the CycT1 mutants lacking its interaction with Tat lost such dynamic movement, this structural fluttering at the Tat-binding cavity involving 10 TRR amino acid residues might be required for stable interaction with Tat. Although Goh et al. proposed three distinct mechanisms for the protein-protein interaction including (i) lock and key model; (ii) induced-fit model; and (iii) preexisting equilibrium model [40], our results with MD simulation favors the preexisting equilibrium model although we cannot exclude the possibility that Tat-CycT1 interaction follows the “induced-fit” model. Therefore, these two interaction models are not mutually exclusive. In our study, MD simulation with WT CycT1 has revealed two successive states, namely “Area 1” and “Area 2” (Fig. 3). Since the Tat-binding pocket of the Area 2 structure, obtained in the absence of Tat, appears to be a reminiscent of the original crystal CycT1 structure (3MI9) (Fig. 4b, see the structural alignment of TRR), our observations of MD simulation for CycT1 molecule fit the concept of “preexisting equilibrium model”. In contrast, in mutant CycT1 proteins, MD simulation exhibited only one major structural model.

In addition, we have identified two distinct intra-molecular hydrogen bonding within CycT1, Q46:Q56 and N60:H183 that were crucial for the CycT1-Tat binding and thus the Tat-mediated HIV transactivation. Although our previous report [20] has described the indispensability of Q46 and H183 in supporting Tat activity, the molecular bases of their indispensability has not been given until this study. Verstraete et al. [41] have revealed Y175 of CycT1 was crucial for the interaction with HEXIM1 and Tat. Similarly, CycT1 V107 was shown to be involved in the CycT1–7SK RNA [42]. Additionally, recent report by Mbonye et al. [43] applied molecular modeling procedure in analyzing the trimolecular structure of CycT1-Tat-Cdk9 (3MI9) and identified S175 of Cdk9 as a crucial AA for the Tat-action. Application of MD simulation together with wet experiments with CycT1 mutants should be useful to further elucidate the molecular mechanism. Collectively, structure-based analyses of protein-protein interaction together with bioinformatics analyses such as MD simulation should be effective in further understanding of biological phenomena in which major factors are identified. Moreover, elucidation of small molecular compounds based on such analyses could further facilitate the development of novel drug therapy.

When we see the status of current chemical therapies against HIV-1, development of Tat inhibitor is desperately needed. Although a number of anti-retroviral agents have proved highly effective, when used in combination, in reducing viral load and delaying disease progression in infected individuals, rapid emergence of the drug-resistant HIV clones due to the error-prone viral life cycle steps in the viral life cycle is inevitable

There are several reports on the identification of small-molecule inhibitors of HIV-1 transcription, such as CDK9 inhibitors and Tat inhibitors [44,45,46]. However, these compounds cannot be used in a clinical setting mostly because of the low therapeutic efficacy and high frequency of unwanted side effects.

Analyses of molecular flexibility during the CycT1-Tat interaction should provide further insights for the understanding of this protein-protein interaction at atomic level to molecular design better fitting inhibitors. Thus, our findings demonstrated in this study should help the development of novel anti-Tat compound. Anti-viral drugs that target cellular cofactors without causing cellular toxicity could be developed based on fine tuning of the target 3D structure and its dynamics. Such compounds, in combination with known anti-HIV compounds, could prevent the progression of HIV-1 infection without the emergence of drug-resistant HIV clones.
